# Increase in body weight is lowered when mice received fecal microbiota transfer from donor mice treated with the AT_1_ receptor antagonist telmisartan

**DOI:** 10.3389/fphar.2024.1453989

**Published:** 2024-11-18

**Authors:** Marco L. Freschi, Axel Künstner, Gianna Huber, Ines Stölting, Hauke Busch, Misa Hirose, Walter Raasch

**Affiliations:** ^1^ Institute of Experimental and Clinical Pharmacology and Toxicology, University of Lübeck, Lübeck, Germany; ^2^ Medical Systems Biology Group, Institute of Experimental Dermatology, University of Lübeck, Lübeck, Germany; ^3^ DZHK (German Centre for Cardiovascular Research), Partner Site Hamburg/Kiel/Lübeck, Lübeck, Germany; ^4^ CBBM (Center of Brain, Behavior and Metabolism), Lübeck, Germany; ^5^ Institute of Experimental Dermatology, University of Lübeck, Lübeck, Germany

**Keywords:** obesity, renin-angiotensin aldosterone system (RAAS), telmisartan, microbiota transfer, desulovibrio, weight reduction, AT_1_ receptor antagonist

## Abstract

**Introduction:**

Treatment of rodents with the AT_1_ blocker (ARB) telmisartan (TEL) has an anti-adipose effect. Among other mechanisms, we also have attributed the anti-adipose action to diet-independent alterations in gut microbiota. Thus, we aimed here to confirm this mechanism by using the fecal microbiota transfer (FMT) approach.

**Methods:**

Seven weeks after initiating a high-fat diet (HFD), C57BL/6N mice received fecal microbiota for 8 weeks from donor mice by oral gavage, continuing HFD feeding. Stool samples came from mice that were treated with TEL (8 mg/kg/d by gavage, 12 weeks), thus remaining lean despite HFD feeding (BL/6>f^TEL^), while controls received feces samples from vehicle/HFD-treated obese mice (BL/6>f^VEH^). Microbiota of the stool samples from these acceptor mice was analyzed by 16S rRNA gene amplicon sequencing.

**Results:**

Weight gain was lower in BL6>f^TEL^ than in BL6>f^VEH^ mice after 3 but not 8 weeks. Energy homeostasis, insulin sensitivity, and body composition did not differ between the two groups. β-diversity indicated group differences (F = 2.27, p = 0.005). Although the Firmicutes/Bacteroides ratio did not differ, abundances of distinct phyla, families, and genera varied. Among others, Ruminococcaceae and Desulfovibrionaceae, Desulfovibrionia uncl., and Lachnospiraceae uncl. were lower in BL/6>f^TEL^ than in BL/6>f^VEH^ mice. Moreover, the correlation between body weight and Lachnospiraceae, Desulfovibrionaceae, Desulfovibrionia uncl., or Desulfovibrio was positive in BL/6>f^VEH^ and negative in BL/6>f^TEL^ mice.

**Discussion:**

As FMT from TEL-pretreated mice influences the microbiota in acceptor mice with slight weight-reducing effects, we confirm the relevance of TEL-related microbiota changes for weight reduction, most likely independent of the transferred stool-residual TEL effect on the host metabolism.

## Introduction

Obesity is a major global health problem (NCD Risk Factor Collaboration NCD-RisC, 2024) and, owing to the higher risk of cardiometabolic complications, it is associated with a sharp decline in life expectancy (Engin, 2017). As lifestyle modifications mostly fail, pharmacotherapy provides an important option for adults who are overweight and obese to reduce their body weight. Although phentermine-topiramate and GLP-1 receptor agonists proved to be best in reducing weight (Shi et al., 2024), it would be desirable to have further drugs with anti-adipose potential for everyday therapeutic practice. This means either that new drugs such as tirzepatide (Frías et al., 2021) or retatrutide (Jastreboff et al., 2023) would need to be approved or established drugs that have an additional, anti-adipose pleiotropic effect be repurposed. In this regard, AT_1_ receptor blockers (ARBs) such as candesartan or telmisartan (TEL), which are well-established in the treatment of hypertension and heart failure, particularly concerning their cardiometabolic benefits (Michel et al., 2016), have been demonstrated several times to lead to a reduction in body weight in rodents (Muller-Fielitz et al., 2012; Muller-Fielitz et al., 2011) and humans (Kintscher et al., 2007). This outcome appears to be a class effect because several ARBs, including candesartan, irbesartan, and TEL, have been shown to reduce body weight (Muller-Fielitz et al., 2012; Muller-Fielitz et al., 2011; Kintscher et al., 2007; Gustaityte et al., 2019; Miesel et al., 2012; Muller-Fielitz et al., 2014; Muller-Fielitz et al., 2015; Nickel et al., 2022; Rawish et al., 2020; Schuchard et al., 2015; Schuster et al., 2018). Interestingly, ARBs reduce body weight more effectively than other pharmacological interventions of the renin-angiotensin system, namely, inhibitors of the angiotensin-converting enzyme (ACE) (Miesel et al., 2012). Moreover, this anti-adipose effect is independent of the blood pressure-lowering effect of these substances (Muller-Fielitz et al., 2014). Concerning weight regulation, we have shown show that the prophylactic or curative treatment of rats and mice with an ARB prevents or reduces the development of diet-induced obesity (DIO) (Muller-Fielitz et al., 2012; Muller-Fielitz et al., 2014). From a mechanistic point of view, this effect is associated with a) an improvement/normalization of leptin sensitivity despite the administration of a cafeteria diet (CD), whereas DIO mice or rats developed leptin resistance (Muller-Fielitz et al., 2011; Muller-Fielitz et al., 2015; Schuster et al., 2018)and/or b) an activation of the ACE2/Ang (NCD Risk Factor Collaboration NCD-RisC, 2024; Engin, 2017; Shi et al., 2024; Frías et al., 2021; Jastreboff et al., 2023; Michel et al., 2016; Muller-Fielitz et al., 2012)/Mas pathway (Schuchard et al., 2015; Blanke et al., 2015; Dapper et al., 2019), with c) cerebral mechanisms also playing a role (Rawish et al., 2020). In contrast, a reduction in stress reactions, which can also contribute to the development of obesity, appears to play a subordinate role (Gustaityte et al., 2019).

In 2006, it was shown for the first time that intestinal bacteria differ in obesity. These results showed an increase in the Firmicutes/*Bacteroides* (F/B) ratio in obese humans and rodents, although it should be noted that the pattern of microbial structure associated with obesity is not necessarily constant, and there are also discrepant reports of no change or even a decrease in the F/B ratio (Fujisaka et al., 2023). A recent study from our group also indicated an influence of TEL on the gut microbiota, suggesting that this effect contributes to its anti-adipose efficacy (Beckmann et al., 2021). The F/B ratio and the abundance of *Blautia*, *Allobaculum*, and *Parasutterella* were higher in DIO controls, but not in TEL-fed rats. Confirming our findings, an increased F/B ratio (Stephens et al., 2018) and higher abundances of *Blautia* (Ottosson et al., 2018; Wang et al., 2005; Lin et al., 2019; Zhang et al., 2019), *Allobaculum* (Liu et al., 2016), and *Parasutterella* (Henneke et al., 2022) were recognized as typical features of obese individuals. In addition, enterotype (ET)-like cluster analyses (Arumugam et al., 2011), Kleinberg’s hub network scoring, and random forest analyses also showed that TEL induces a specific signature in the gut microbiota (Beckmann et al., 2021). Furthermore, the additional per-fed (PF) group (which was adapted to the dietary intake of the TEL group) confirmed that the anti-adipose effect of TEL treatment was due to diet-independent changes in the gut microbiota, as the microbiota of TEL-treated rats that were fed with CD differed significantly from the microbiota of DIO and PF rats (Beckmann et al., 2021). We further confirmed the gastrointestinal potential of TEL as it influenced the intestinal mucosal thickness in obese mice (Nickel et al., 2022). On the basis of preliminary data, we had already published the results of fecal microbiota transfer (FMT) in mice to strengthen the influence of TEL on the microbiota (Beckmann et al., 2021). However, that publication lacked detailed evaluations regarding phylum, family, and genus and, in particular, the respective effect sizes and, in addition, their correlations to body weight. Therefore, the aim of the present study was to resolve the issue of missing information. Such knowledge would be important in the follow-up of further mechanistic studies on the relationship between the microbiome and TEL.

## Methods

### Animals

The animal experiments were conducted according to the NIH guidelines for the handling of laboratory animals. The experiments were approved by the *Ministerium für Energiewende, Landwirtschaft, Umwelt, Natur und Digitalisierung des Landes* Schleswig-Holstein, Germany (experiment number: 86–9/18). For the experiments, 24 male C57BL/6N mice from breeder Charles River were used. The animals were 6 weeks old on delivery and had a body weight of 22.0 g (SD = 1.1 g). Two weeks before the start of the experiment, the animals were adapted to the housing conditions. Four mice were housed in individually ventilated cages (IVC) with an area of 500 cm^2^ at a constant temperature of approximately 21°C, a humidity of 40%–60%, and a 12-h day (6 a.m.–6 p.m.) - night cycle (6 p.m.–6 a.m.).

### Protocol

(See Supplementary Figure S1): At the beginning of the experiment, the mice were randomly assigned to one of two experimental groups of 12 individuals. Mutual influence on the intestinal flora of animals living together is already known (Ivanov et al., 2008); therefore, only animals from the same experimental group were housed in each cage. The animals had free access to food and drinking water for the entire duration of the experiment. At the start of the experiment, the test groups were given a high-fat diet (HFD). The diet (60% KJ Fat Lard) from SSNIFF Spezialdiäten GmbH has a nutritional value of 25 kJ/g (60% fat, 20% protein, and 20% carbohydrates). All animals were weighed three times a week for the entire duration of the experiment. From day (d) 49 until d101, the FMT took place. The donor stool samples were obtained in a previous study (Huber et al., 2021). Donor mice were HFD-fed C57BL/6N mice that had been treated for 12 weeks either with vehicle (VEH) or TEL (8 mg/kg/d, by gavage, 12 weeks). The body weight of the two groups of donor mice did not differ at the start of the study (19.5 ± 2.3 vs 19.8 ± 2.3 g), but the VEH-treated donor mice developed manifest obesity, while the TEL-treated donor mice remained protected from this (48.4 ± 3.2 vs 34.8 ± 2.6 g, P< 0.0001) (Huber et al., 2021). The feces of these donor mice were collected within 1 h after excretion and frozen at −80°C immediately afterward. After FMT (for detailed description, see below), the animals were subjected to laboratory-established functional tests during the intervention, namely, indirect calorimetry at d70-d77 using the Phenomaster System (TSE, Germany) (Rawish et al., 2020), body composition measurement 2 h after the mice were transferred to the room to acclimatize to the environment by using the Minispec BCA analyzer (LF-110, Bruker) (at d92) (Huber et al., 2021; Hernandez Torres et al., 2024), and an insulin tolerance test (ITT) at d84 (Rawish et al., 2020; Dapper et al., 2019). The ITT was performed on 8-h fasting mice by administering 1 IU insulin/kg_b.w._ Intraperitoneally (i.p.) and drawing blood from the tail tip after 0, 20, 40, 60, 80, and 120 min to determine glucose levels using a commercial glucose sensor (Accu Chek Inform II strips and Accu Chek Performav blood glucose meter, Roche, Germany). At the end of the 8-week FMT period, the animals were killed and the organs removed. Stool samples were collected at three time points for microbiota analysis: 0, 49, and 101 days. The samples at d0 and d49 were collected from the cages and thus represent the pooled microbiota of four experimental animals. The samples at the end of the experiment at d101 were taken directly from the colon at the time of killing and represent the specific microbiota of a single animal.

Fecal microbiota transfer: The acceptor mice had not been pretreated with antibiotics prior to the FM. The frozen stool samples were prepared for microbiome transfer according to an established protocol (Ellekilde et al., 2014). The stool samples were homogenized 1:20 in a 50% glycerol/phosphate-buffered saline (PBS) solution (Carl Roth) using an ultra tourax homogenizer. The resulting suspension was filtered through a 5-µm syringe filter (Minisart, Sartorius) and afterward aliquoted in 150-µL portions frozen at −20°C. The resulting aliquots were diluted 1:5 with 50% glycerol/PBS solution (Carl Roth) shortly before use. Regardless of body weight, each animal was administered 150 µL of the suspension by gavage 3 days per week (Monday, Wednesday, Friday) for 8 weeks (Supplementary Figure S1).

### DNA extraction and 16s rRNA gene sequencing of mouse stool samples

Bacterial DNA extraction, library sequencing for the 16S rRNA gene were performed as previously described (Beckmann et al., 2021). In brief, bacterial DNA samples were prepared from the stool samples using the Qiagen PowerSoil Kit (QIAGEN GmbH, Hilden, Germany), according to the manufacturer’s protocol. The hypervariable V3 and V4 region of the 16S rRNA gene was amplified using polymerase chain reaction (PCR) and a 341F/806R primer combination, employing a dual-index strategy. The PCR products were pooled into equimolar subpools and subsequently purified by gel extraction and magnetic beads. The quality of the final library was determined by using an Agilent 2100 Bioanalyzer, and the concentration was quantified using the NEBNext Library Quant Kit for Illumina (New England BioLabs), according to the manufacturer’s instructions. The final library was sequenced on the Illumina MiSeq platform using v3 chemistry (600 cycles, Illumina Inc. San Diego, CA, United States).

### Processing and analysis of DNA sequencing

Sequencing reads were filtered for quality using VSEARCH (vsearch v2.12.0; https://github.com/torognes/vsearch-eval) (Rognes et al., 2016). The artifacts (chimeras) resulting from incorrect fusion during PCR were detected and eliminated using the corresponding VSEARCH tool, with data from RDP Gold V9 (http://rdp.cme.msu.edu/misc/rel10info.jsp#taxonomy) serving as a reference. To counteract false low differences in the prevalence of a certain species (Evans and Denef, 2020), dereplication was performed using VSEARCH. With version V11.0.667 of USEARCH, so-called operational taxonomic units (OTUs) could be formed from the prefiltered data. Potential contaminations of the samples were filtered out with the tool decontam (1.8.0, https://bioconductor.riken.jp/packages/3.11/bioc/html/decontam.html).

The alpha diversity taking into account the number of species was calculated as Shannon diversity using DivNet (0.3.6) and the confidence interval was displayed as a violin plot (Konopiński, 2020). The significance of the differences was determined using a Wilcoxon test. Beta diversity was assessed by calculating the Aitchison distance. The differences in the abundance of the various phyla, families, and genera were described with the tool ANCOMBC (0.99.4, https://www.bioconductor.org/packages/release/bioc/vignettes/ANCOMBC/inst/doc/ANCOMBC.html) using differential abundance analysis (DAA). The effect size between taxonomic abundance was calculated by using http://www.statistik-beratung.net/?Effektstaerke-Rechner.

### Data evaluation and statistics

The change in glucose in the ITT was statistically analyzed using the area under the curve (AUC), taking into account the delta values relative to the baseline glucose value. The statistical analysis of the data was carried out using the Prism 8 program (GraphPad, San Diego, United States). The values were tested for normal distribution and homogeneity of variance before t-testing or ANOVA testing. The t-test was used to compare two groups. If there was no variance homogeneity, the t-test was corrected according to Welch. When two factors were to be tested, a 2-way analysis of variance (ANOVA) was used. Differences were considered statistically significant if the calculated *p*-value was less than 5%. In figures, means±SDs are depicted. Correlation analyses were performed by a 2-tailed Pearson test.

## Results

### Upon FMT with stool samples from donor mice pretreated with TEL, the body weight of the acceptor mice is slightly reduced

**FIGURE 1:**
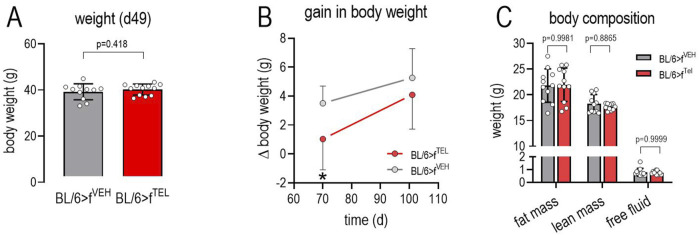
In FMT with fecal samples from donor mice that were pretreated with TEL, the body weight of the recipient mice is slightly reduced. **(A)** body weight at d49 before starting FMT; **(B)** weight gain in BL/6>f^VEH^ or BL/6>f^TEL^ mice during the FMT; 2-way ANOVA indicates increase in dependency of time and difference between the two groups (time: F = 9.381, *p* = 0.0039; FMT: F = 16.45, *p* = 0.0002; Interaction: F = 1.193, *p* = 0.2813); Sidak’s multiple comparisons test specified significant reduction in weight gain at d70; **(C)** body composition determined at d92; means ± SD; n = 11–12.

**FIGURE 2:**
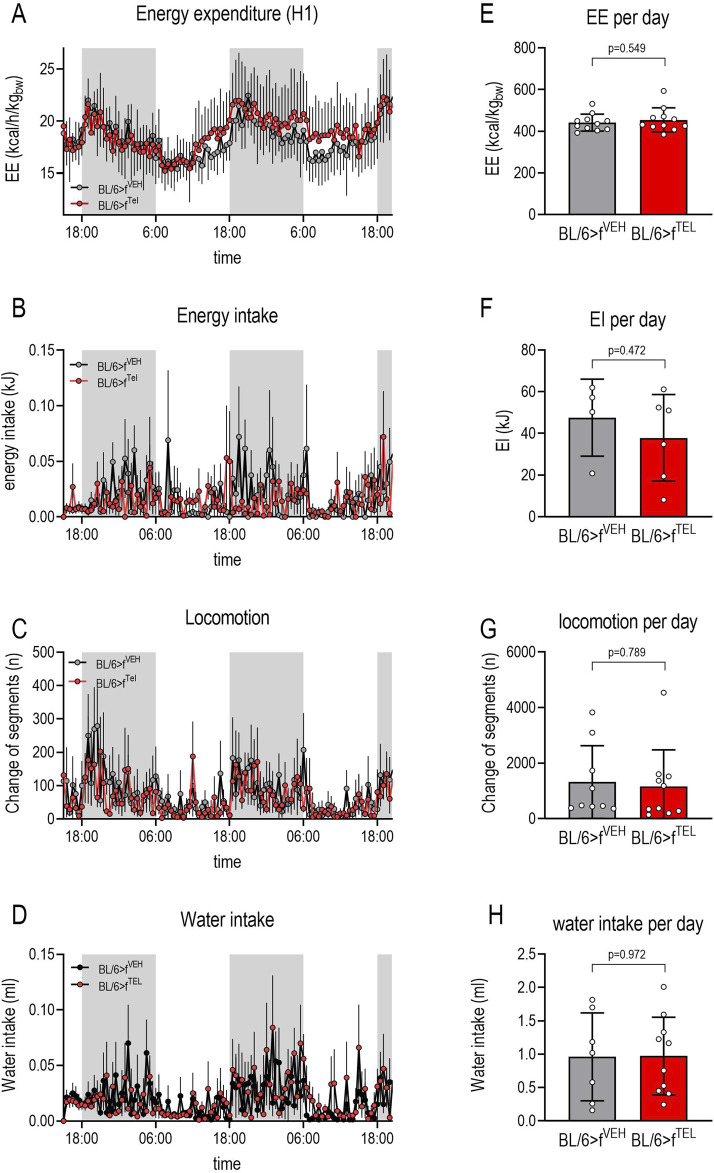
Energy homeostasis did not differ between BL/6>f^VEH^ and BL/6>f^TEL^ mice: Following 2-way ANOVA, a day/night rhythm was observed for energy expenditure [**(A)**: F = 12.07, P< 0.0001], energy intake [**(B)**: F = 21.38, *p* = 0.0009], locomotion [**(C)**: F = 2.643, *p* = 0.0425], and water intake [**(D)**: F = 10.33, P< 0.0001] in both treatment groups, while the FMT had no effect. This equality between the two groups was further confirmed when the cumulative energy consumption **(E)**, energy intake **(F)**, locomotion **(G)**, and water intake **(H)** over a day were taken into account (means ± SD, group size 11–12); however, in some cases only 4 animals could be taken into account, particularly in terms of energy and water intake, as some of the mice were unable to feed themselves adequately from the food baskets or drinking bottles.

At the start of the study, the weight of the two groups did not differ (22.3 ± 1,1 vs 22.7 ± 1.2 g). As a result of feeding the HFD, body weight increased in both groups until d49 without any difference (Figure 1A). A further weight increase was also observed in the BL/6>f^VEH^ until d70, whereas this was less frequently observed in BL/6>f^TEL^ mice, resulting in a significantly lower weight gain until d70 (Figure 1B). However, the feeding behavior of the mice was affected by calorimetric testing, which was carried out following d70 to d77, such that some of the mice did not eat the food from the food baskets and did not drink from the bottles, which led to increased rebound eating and thus also had a lasting effect on body weight. Due to this increased food intake, the weight gain did not also differ at d101 between the two experimental groups (Figure 1B). On d96, the mice were analyzed for body composition and showed no differences in fat mass, lean mass, and free body fluid between the BL/6>f^VEH^ and BL/6>f^TEL^ mice (Figure 1C). Using the Phenomaster System we could indeed confirm a circadian rhythm of energy expenditure, energy and water intake, as well as in locomotion; however, we failed to detect any differences between the two mouse groups (Figure 2). In addition, we calculated the quotient between daily energy consumption and daily energy intake. This indicates that energy consumption relative to energy intake tends to be higher in the BL/6>f^TEL^ mice (Supplementary Figure S2). However, this result must be evaluated with caution, especially as the sample size in the control group is too small at n = 4 due to the feeding problems in the Phenomaster system (see above) and the statistical evaluation is therefore limited.

### FMT with stool samples from donor mice pretreated with TEL does not affect glucose control of the acceptor mice

**FIGURE 3:**
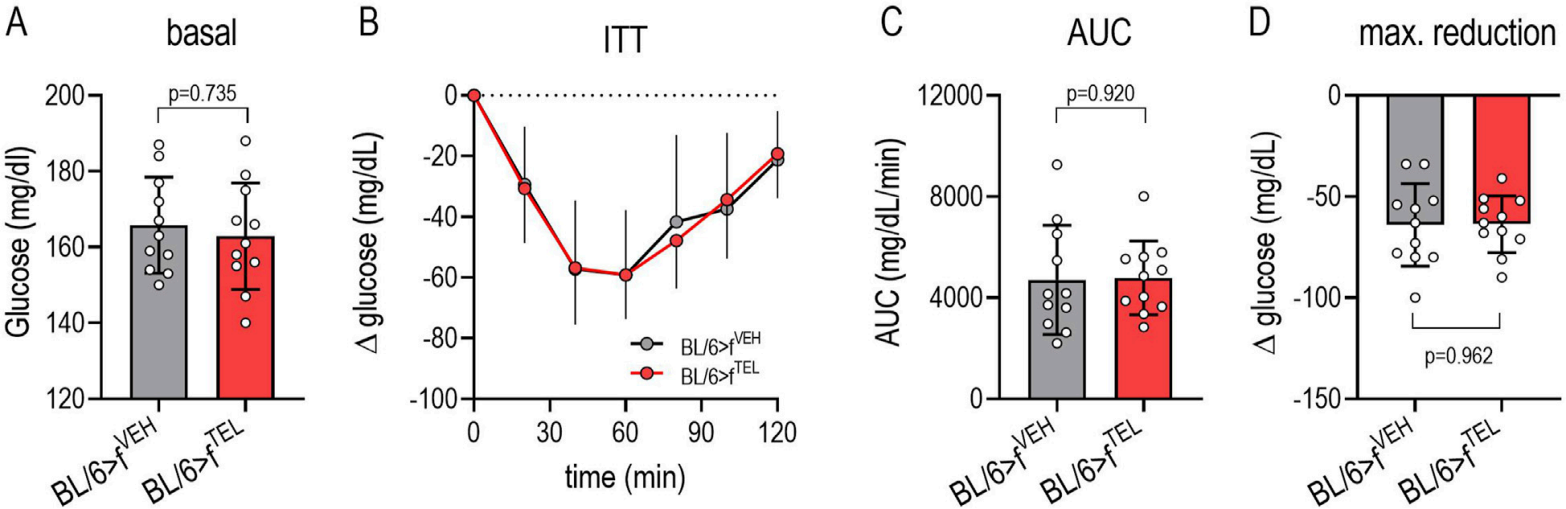
FMT with fecal samples from donor mice pretreated with TEL has no effect on blood sugar control in recipient mice: **(A)** Shows the fasting blood glucose values of mice before starting the ITT. **(B)** Shows the change in blood glucose after insulin injection. The 2-way ANOVA analysis indicates a dependence on time for both groups (F = 58.32, *p* < 0.0001), but no influence of the different FMTs (F = 0.0024, *p* = 0.961); this is confirmed as AUCs **(C)** and maximal glucose reduction **(D)** did not differ between the two groups; MW±SD n = 11; a t-test was used to statistically compare two groups.

The fasting blood glucose levels at the beginning of the ITT did not differ between the two groups (Figure 3A). As a result of the insulin injection, blood glucose decreased within 1 h to minimal values (52.7 ± 13.5 vs 61.6 ± 18.9 min, *p* = 0.208, Figure 3B), whereby neither the maximum reduction (Figure 3D) nor the AUC (Figure 3C), which represents the total extent of the insulin-dependent change, differed between BL/6>f^VEH^ and BL/6>f^TEL^ mice.

### Microbiota differs between BL/6>f^TEL^ and BL/6>f^VEH^ mice

**FIGURE 4:**
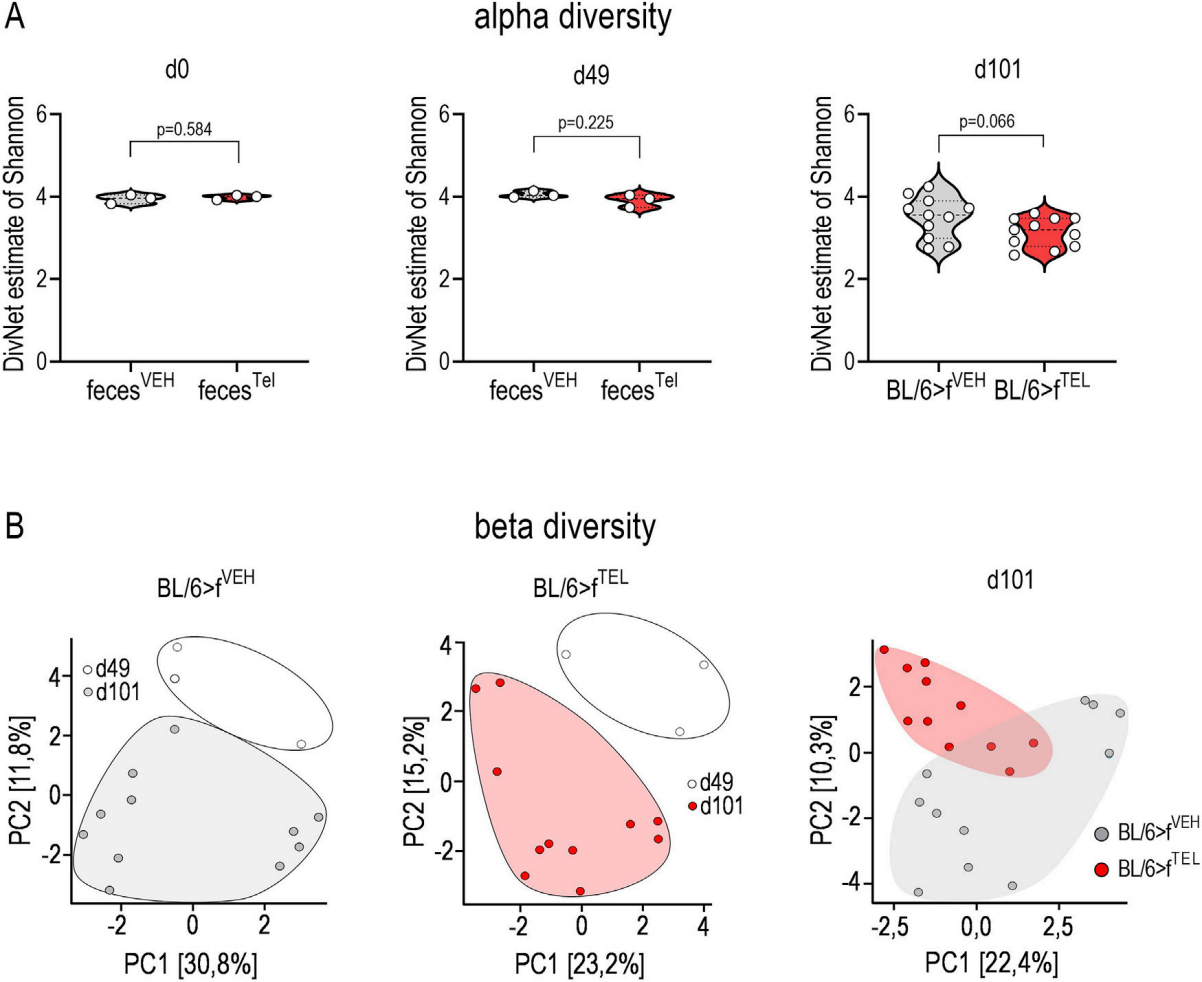
Beta diversity, but not alpha diversity is affected when recipient mice received stool samples from donor mice pretreated with TEL. **(A)** Alpha diversity at day 0, 49, and 101 comparing the two collectives, BL/6>f^VEH^ and BL/6>f^TEL^. **(B)** Beta diversity according to Aitchison, showing the comparison between d49 and d101 of the BL/6>f^VEH^ mice (F = 1.426, *p* = 0.095) and BL/6>f^TEL^ group (F = 2.34, *p* = 0.008) as well as the comparison between BL/6>f^VEH^ and BL/6>f^TEL^ at d101 (F = 2.27, *p* = 0.005). The transplantation of the stool samples took place at d49 immediately after stool collection. At days 0 and 49, the mice were in groups of 4 animals each in the cages. The stool samples were collected from the cages. Therefore, one data point representing d0 and49 summarizes 4 mice. In contrast, at day 101, feces were collected individually from the colon. Statistical comparison was done using an unpaired t-test.

**FIGURE 5:**
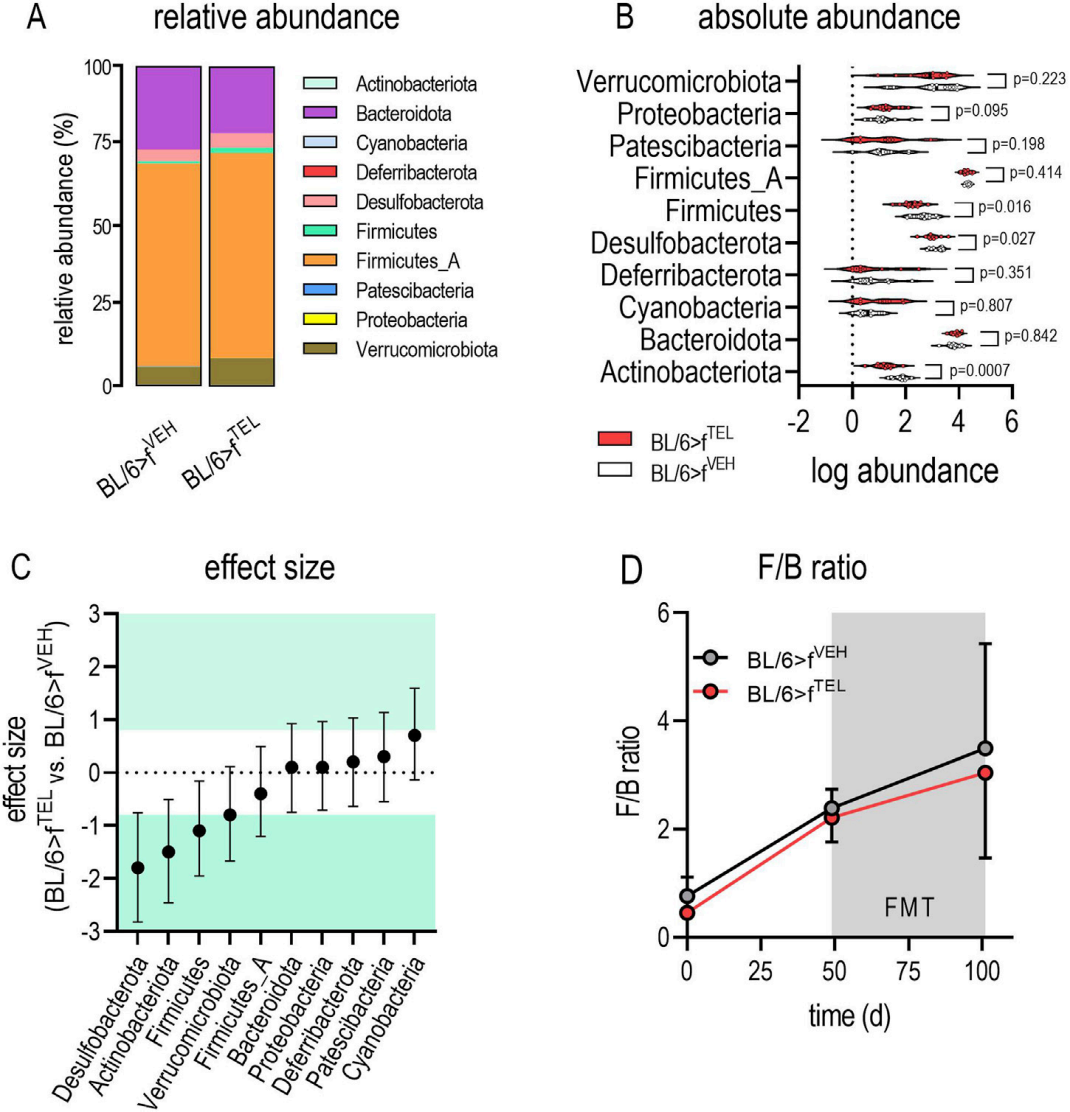
Phylum abundance differs in feces of BL/6>f^VEH^ and BL/6>f^TEL^ mice. **(A)** relative abundances; **(B)** absolute abundances; the statistical comparison was made with an unpaired t-test when values were Gaussian distributed (considering Welch correction when variances differed) or by using Mann-Whitney test as a nonparametric test; **(C)** Cohen’s effect size, which was classified as small (d = ±0.2), medium (d = ±0.5), and large (d ≥ ±0.8, green colored; http://www.statistik-beratung.net/?Effektstaerke-Rechner); **(D)** F/B ratio over time for the two experimental groups (2-way ANOVA: time F = 7.654, *p* = 0.0023, TEL F = 0.2580, *p* = 0.6156; interaction F = 0.02135, *p* = 0.9789); means ± SD, n = 11–12.

**FIGURE 6:**
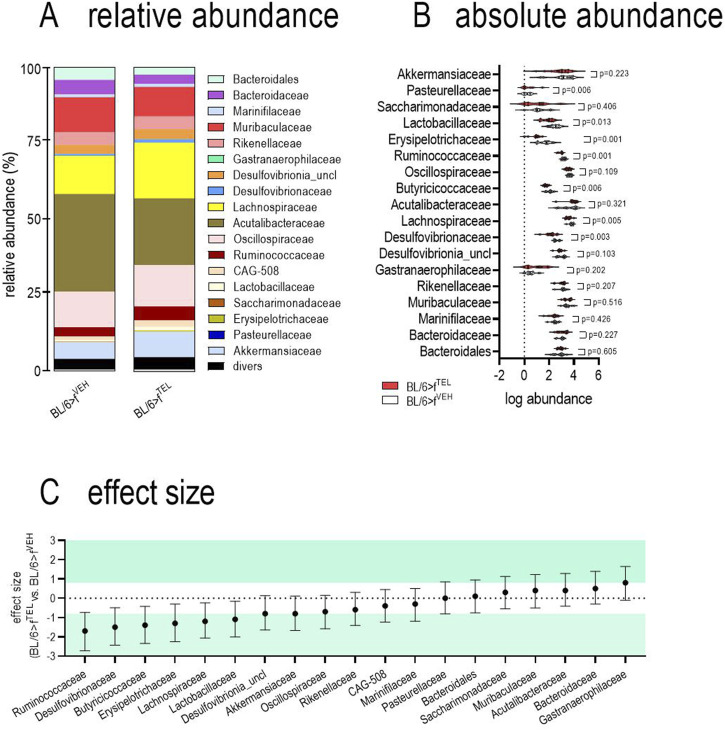
Family abundance differs in feces of BL/6>f^VEH^ and BL/6>f^TEL^ mice. **(A)** relative abundances; **(B)** absolute abundances; the statistical comparison was made with an unpaired t-test when values were Gaussian distributed (considering Welch correction when variances differed) or by using Mann-Whitney test as a nonparametric test. **(C)** Cohen’s effect size, which was classified as small (d = −0.2–0.2), medium (d −0.8–0.8), and large (d ≥ 0.8, green colored; http://www.statistik-beratung.net/?Effektstaerke-Rechner); means ± SD, n = 11–12.

**FIGURE 7:**
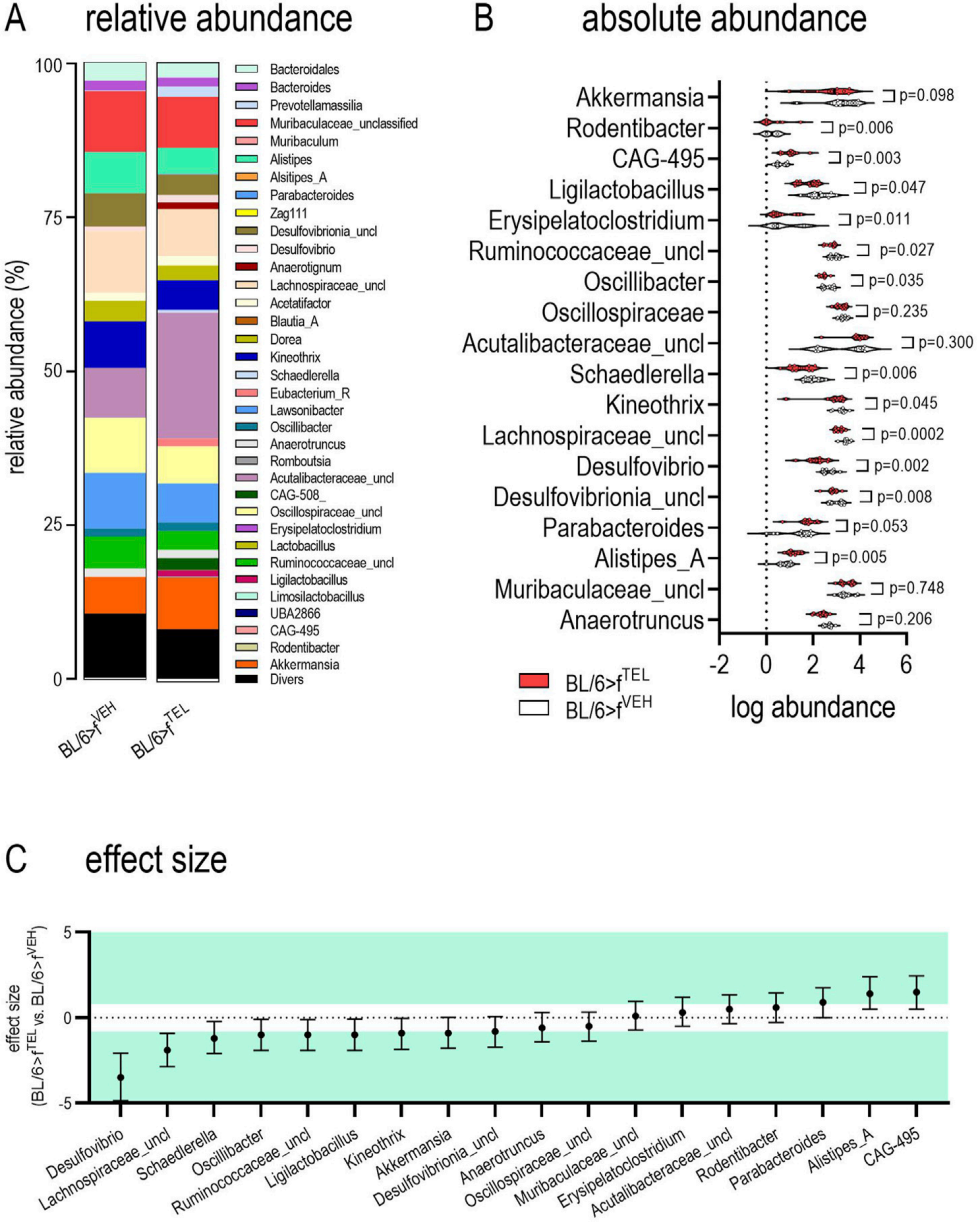
Genus abundance differs in feces of BL/6>f^VEH^ and BL/6>f^TEL^ mice. **(A)** relative abundances; **(B)** absolute abundances; the statistical comparison was made with an unpaired t-test when values were Gaussian distributed (considering Welch correction when variances differed) or by using Mann-Whitney test as a nonparametric test. **(C)** Cohen’s effect size, which was classified as small (d = 0.2), medium (d = 0.5), and large (d ≥ 0.8, green colored; http://www.statistik-beratung.net/?Effektstaerke-Rechner); means ± SD, n = 11–12.

**FIGURE 8:**
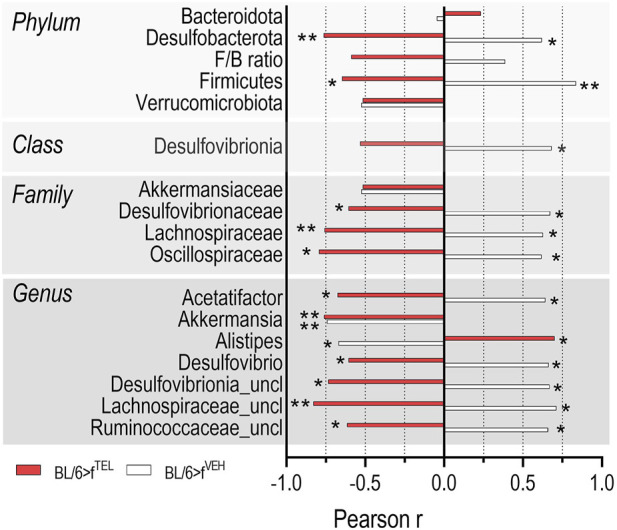
The correlation between body weight and the abundance of certain phyla, families, and genera is regulated in opposite ways when recipient mice received stool samples from donor mice pretreated with TEL or vehicle (for correlation plots see Supplementary Figures S4-S6). The correlation analyses were carried out using Prism 8.0.

At time points d0 and d49, taxonomic abundances of phyla (Supplementary Figure S3) and alpha diversity did not differ between the two groups (Figure 4A). At time d101, at the end of the trial, alpha diversity did not quite reach the level of significance (*p* = 0.066) between the BL/6>f^TEL^ and the BL/6>f^VEH^ mice (Figure 4A. To further evaluate effects on gut microbiota, beta diversity was calculated according to Aitchinson. Taxonomic changes could be observed depending on the HFD diet, as the abundances of phylum, family, and genus differed very clearly between d0, when the mice were still fed chow diet, and those at d49, after the mice had already been fed HFD for 7 weeks (Supplementary Figures S3–S6). As a result, the F/B ratio was also higher on d49 than on d0 (Figure 5D). Sorted by decreasing effect size, at the genus level, the abundances of *Duncaniella*, *Turicimonas*, and *Paramuribaculum* in particular were considerably lower at d0 than at d49 (Cohens d < -2), but, conversely, those of *Lawsonibacter*, *Alistipes*, *Oscillospiraceae uncl.*, *Ruminococcaceae uncl.*, *Oscillibacter Ligilactobacillus Anaerotignum Acetatifactor*, *Desulfovibrionia uncl.*, *Kineothrix Bacteroidales*, *Parabacteroides*, and *Faecalibaculum* (Cohens d > 2) were noticeably higher at d49 than at d0 (Supplementary Figure S5; Supplementary Table S1). In contrast to BL/6>f^VEH^ (F = 1.426, p = 0.095), BL/6>f^TEL^ mice (F = 2.34, *p* = 0.008) showed a clear change in beta diversity as a result of FMT when d49 was compared with d101 (Figure 4B), although the different collection sites of the stool samples at d49 and d101 could also contribute to these differences. In addition, beta diversity was different between BL/6>f^VEH^ BL/6>f^TEL^ mice at d101 (F = 2.27, p = 0.005; Figure 4B). Since beta diversity suggested differences in gut microbiota in response to fecal transfer with feces samples from TEL-pretreated individuals compared to FMT’s VEH-treated feces samples, we next analyzed taxonomic abundances. At the phylum level, we identified a decreased abundance of *Actinobacteriota*, *Desulfobacterota*, and *Firmicutes* in the BL/6>f^TEL^ mice. It should be noted that the relative abundance of each of these three phyla was less than 5%. (Figure 5A, B). The influence of TEL on phylum level was also confirmed in the effect size calculation (Figure 5C). However, the F/B ratio was not decreased in the BL/6>f^TEL^ compared to BL/6>f^VEH^ mice as expected, at least at d101 (Figure 5C, D): here, the correlation between the F/B ratio and body weight is regulated in opposite ways, but Pearson r did not reach the significance level of 0.05 (Figure 8; Supplementary Figure S6). At the family level, the abundance was significantly lower for *Erysipelotrichaceae* (−87%), *Lactobacillaceae* (−70%), *Ruminococcaceae* (−43%), *Butyricicoccaceae* (−53%), *Lachnospiraceae* (−37%), and *Desulfovibrionaceae* (−57%) (Figure 6A, B). Remarkably, the abundance of *Lachnospiraceae* is relatively high here, at almost 20% in the control mice, while this rate was usually between 1% and 5% in the other families mentioned. Only *Pasteurellaceae* (+975%) was higher in BL/6>f^TEL^ mice, although their abundance was low. TEL effects were again reflected by a medium (Cohen’s d > 0.2, <0.5) to larger effect size (Cohen’s d > 0.5, Figure 6C). The abundance of the genera *Ligilactobacillus* (−72%), *Ruminococcaceae uncl.* (−36%), *Oscillibacter* (−37%), *Schaedlerella* (−67%), *Kineothrix* (−40%), *Lachnospiraceae uncl.* (−48%), *Desulfovibrio* (−66%), *Desulfovibrionia uncl.* (−35%) was lower in the BL/6>f^TEL^ mice than in the BL/6>f^VEH^ mice while *Rodentibacter* (+975%), *CAG*-495 (+272%), *Erysipelatoclostridium* (+96%), and *Alistipes_A* (+153%) were higher. However, it should be noted that the relative abundance of the genera that were downregulated in the BL/6>f^TEL^ mice was <5% in BL/6>f^VEH^ mice, except for *Lachnospiraceae uncl.* With a relative abundance of about 7%. The relative abundance of those genera that were upregulated in the BL/6>f^TEL^ mice was very low at <0.1%. TEL-induced regulation was also reflected in altered effect size (Figure 7). Interestingly, significant correlations were observed in the correlation analyses between the body weight of the mice and the microbiota. At the phylum level, a positive correlation was observed for Desulfobacterota and Firmicutes in BL/6>f^VEH^ mice, which was reversed to negative values in the BL/6>f^TEL^ mice (Figure 8; Supplementary Figure S5). At the family level we also observed in BL/6>f^VEH^ mice a positive correlation between body weight and *Oscillospiraceae, Lachnospiraceae, and Desulfovibrionaceae*, respectively, which reverted to almost negative values in the BL/6>f^TEL^ mice (Figure 8; Supplementary Figure S6). At the genus level the correlation between body weight and *Ruminococcaceae uncl., Lachnospiraceae uncl., Desulfovibrionia uncl., Desulfovibrio, and Acetatifactor*, respectively, were negative in the BL/6>f^VEH^ and positive in the BL/6>f^TEL^ mice, while positive in BL/6>f^VEH^ and negative in the BL/6>f^TEL^ mice considering the correlation between body weight and *Alistipes*. Strikingly, in both groups, the correlation was negative between body weight and *Akkermansia* (Figure 8; Supplementary Figure S7).

## Discussion

The key finding of the present study was the discrete weight loss in obese mice with concomitant changes in the gastrointestinal microbiota when these mice received feces from donor mice previously treated long term with TEL, while glucose homeostasis of these acceptor mice remained unaffected. Thus, the following discussion focuses on two main aspects: firstly, the possible influence of TEL on the microbiota and, secondly, determining which phyla, families, and genera are regulated by TEL and their mechanistic function in regulating weight.

### Influence of TEL on the microbiota

TEL is largely excreted in the feces in a pure, nonconjugated form, raising the question as to whether remaining TEL residues in the stool samples of the donor mice have direct effects on the microbiota of the acceptor mice (Stangier et al., 2000). Considering the pharmacokinetic properties of TEL and the dilution effects during stool preparation for FMT (for detailed calculations, see Supplementary Data) we extrapolated a daily TEL dose of approx. 0.0016 mg/kg^bw^ by FMT. As anti-adipose effects of TEL were only observed with high-dose therapy (Muller-Fielitz et al., 2011; Muller-Fielitz et al., 2014), which are therefore 5,000 times higher than those administered by FMT, the TEL plasma concentrations potentially absorbed from the gastrointestinal tract would be insufficient to exert a disruptive, direct effect and would not justify the effects expected in the microbiota.

Since direct effects due to TEL contamination in stool samples can be excluded, we suggest that other TEL-dependent mechanisms contribute to the weight loss of the acceptor mice and, according to our hypothesis, we suspect that microbiota-dependent effects are involved. This assumption is supported by studies in obese mice showing that their body weight was reduced and their insulin sensitivity was improved when they received fecal transplants from normal-weight donors (de Groot et al., 2017). Based on our various findings on ARB-induced weight loss (Muller-Fielitz et al., 2012; Muller-Fielitz et al., 2011; Miesel et al., 2012; Muller-Fielitz et al., 2014; Muller-Fielitz et al., 2015; Nickel et al., 2022; Rawish et al., 2020; Schuchard et al., 2015; Schuster et al., 2018; Dapper et al., 2019; Winkler et al., 2018; Winkler et al., 2016) as well as our previous observations that TEL leads to specific changes in the gastrointestinal microbiota that were discussed to contribute to TEL-induced weight loss (Beckmann et al., 2021), we were encouraged to assume that TEL-induced changes in the microbiota may also alter the microbiota of the acceptor animals after stool transfer, which could lead to weight loss. Whereas we failed to analyze the microbiota of the transferred stool samples in the present study, which does represent a limitation, we have already shown in our previous study that the intestinal microbiome was altered by TEL therapy, independent of the dietary intake of fat and sugar. Why the microbiome of the donor mice is altered under TEL is still unclear and we can only speculate about the cause. It is at least conceivable, but unproven, that due to the moderate bioavailability of TEL (approx. 40%) (Sekar and Chellan, 2008) the remaining amount of intestinal TEL changes the intestinal environment in such a way that the growth of certain bacteria is influenced or that TEL itself is used as a metabolic product and thus gives certain bacteria an advantage over others. However, this aspect was not the subject of the present study and therefore it was not discussed further, especially as, to our knowledge, no information on this subject is available.

### Phyla, families, and genera regulated by FMT from TEL-pretreated donor mice

We observed clear changes in the microbiota of the acceptor mice, which in our study were not antibiotic-depleted (Abx). In a review article for FMT experiments on mice, a procedure was recommended for obtaining a germ-free, comparable condition when exploring the pathogenesis and potential treatments for microbiota-related diseases (Shang et al., 2022). Our decision to use non-ABx-primed mice was based on the fact that pretreatment with antibiotics significantly affects the gut microbiota and thus also influences body weight and metabolic parameters (Yu Z. et al., 2022).

Indeed, studies have consistently shown alterations in the composition of gut bacteria in obese individuals, with an increased abundance of Firmicutes and a decreased abundance of Bacteroidetes at the phylum level (Cadena-Ullauri et al., 2024). In the hierarchical ranking of bacterial taxonomy, our results were also conclusive concerning the phylum Firmicutes, with the family *Oscillospiraceae* and the associated genus *Lawsonibacter*, as well as with the associated family *Lachnospiraceae* with the genera *Acetatifactor*, *Kineothrix*, *Lachnospiraceae uncl*., and *Anaerotignum*, as 1.) the abundances of all these bacteria were increased after the 49d HFD feeding period; 2.) *Lachnospiraceae* and *Oscillospiraceae* were also lower in the BL/6>f^TEL^ than in the BL/6>f^VEH^ mice, as were the associated genera *Lachnospiraceae uncl.* And *Kineothrix*; and 3.) body weight and the corresponding abundances of the phylum Firmicutes, the families *Lachnospiraceae* and *Oscillospiraceae*, and the genera *Acetatifactor* and *Lachnospiraceae uncl.* Positively correlated in the BL/6>f^VEH^ mice whereas this correlation was negative in the BL/6>f^TEL^ mice. Moreover, *Erysipelotrichaceae and Faecalibaculum*, which also belong to the Firmicutes, positively correlated with obesity, as they were increased after 49d HFD feeding and at least *Erysipelotrichaceae* were lower in BL/6>f^TEL^ than in the BL/6>f^VEH^ mice. For Firmicutes, we therefore confirm experimental and clinical findings of others showing in mice and humans that *Erysipelotrichaceae,* Oscillospiraceae, and *Lachnospiraceae* were increased (Lamichhane et al., 2024; Huang et al., 2023; Duan et al., 2024; Zhu et al., 2023; Cheng et al., 2022; Hernández-Montoliu et al., 2023), but decreased again upon weight loss (Lamichhane et al., 2024), drug-induced weight reduction using Qingmao/Qingzhuan Tea (Cheng et al., 2022), semaglutide or inulin (Huang et al., 2023; Duan et al., 2024), or following Roux-en-Y gastric bypass surgery (RYGB) (Hernández-Montoliu et al., 2023). The genera *Lawsonibacter, Lachnospiraceae uncl., Acetatifactor, Anaerotignum*, and *Faecalibaculum* were also identified to be higher in mice upon HFD feeding and lower in humans after RYGB (Lamichhane et al., 2024; Zhu et al., 2023; Cheng et al., 2022; Zheng et al., 2024; Prykhodko et al., 2024). Given the established view that Bacteroidetes are reduced in obesity, we confirmed here that at the family level *Muribaculaceae* (Ye et al., 2024) and the genera *Duncaniella* and *Paramuribaculum* are in part very significantly reduced after 49d HFD feeding. As changes at the *Muribaculaceae* level were not confirmed in our FMT experiments, we assume that this family was less or hardly affected. In agreement with literature findings (Li et al., 2024), we discovered that *Turicimonas* was very noticeably reduced upon HFD feeding, whereas *Turicimonas* was not regulated in the FMT experiment. We even found a consistent drop in abundance at phylum level (*Proteobacteria*) from about 5% at d0 to <0.1% at d49, which, however, seems contradictory in light of the finding that *Proteobacteria* was the most consistently reported obesity-associated phylum (Xu et al., 2022).

The most impressive results at the phylum, class, family, and genus level are that, on the one hand, the abundances of *Desulfobacterota*, *Desulfovibrionia*, *Desulfovibrionaceae*, and *Desulfovibrio* consistently increased during the 49 days of HFD feeding, but, on the other hand, reliably decreased when obese mice underwent FMT from TEL-pretreated donor mice. *Desulfovibrio* is ubiquitous in the human gastrointestinal tract as a commensal by playing only a minor role in the healthy gut, but being an opportunistic pathobiont that can be found in various intestinal and extra-intestinal diseases, also including metabolic syndrome (Singh et al., 2023). Our findings on the relationship between abundance and body weight are consistent with the majority of the literature findings. Thus, at the phylum level HFD feeding promoted the growth of the opportunistic pathogen *Desulfobacterota* (Wei et al., 2021; Wang et al., 2022) Conversely, treatment of HFD-fed mice with theabrownin (the most abundant content in Fu Brick tea) or the lipid-reducing drug fenofibrate or soluble dietary fibers (glucomannan-dihydromyricetin complex) not only lowered fat deposition and body weight, but also the abundance of Desulfobacterota (Wang et al., 2022; Lu et al., 2024; Ruan et al., 2023). Below the phylum level, a dependency on obesity was also observed at the family or genus level. At the family level, for example, it was shown that abundance of *Desulfovibrionaceae* was higher in DIO mice (Guerbette et al., 2023), but reduced when obese mice underwent one-anastomosis gastric bypass by leading to a significant loss of body weight (Lazaridis et al., 2024). Confirming recent reports in mice or pigs (Li et al., 2024; Singh et al., 2023; Angoa-Pérez et al., 2020; Yu Y. et al., 2022; Zheng et al., 2022; Panasevich et al., 2018), we moreover confirmed at the genus level that the abundance of *Desulfovibrio* and *Desulfovibrionia uncl.*, respectively, depend on body weight as the abundance of *Desulfovibrio* decreased significantly (−65%, effect size 3.5) and *Desulfovibrio* and body weight of ^BL/6>fVEH^ and ^BL/6>fTEL^ mice correlated positively and negatively with each other (this was also observed for *Desulfovibrionia uncl*, albeit to a lesser extent).

### The role of significantly altered bacterial strains (especially desulfovibrio) for weight loss

The dependency between obesity and *Desulfovibrio* also became apparent in treatment studies as HFD-induced increase of *Desulfovibrio* was lowered in parallel to weight normalizing when mice were treated with probiotics, orlistat, or Zeaxanthin (Wang et al., 2020; Jin et al., 2022; Xie et al., 2021). These findings from animal experiments were confirmed at the human level, as 1.) obese patients as well as T2DM patients also showed a high abundance of *Desulfovibrio* (Qin et al., 2012; Karlsson et al., 2013; Olsson et al., 2020); and 2.) the abundance of *Desulfovibrio* was approximately halved when patients with T2DM were treated with a high-fiber diet (Chen et al., 2023; Fu et al., 2022). Thus, although we reported the cross-talk between *Desulfovibrio* bacteria and body weight regulation in our study, the underlying mechanisms here remain largely unclear. As typical sulfate-reducing bacteria, the effects of *Desulfovibrio* were mostly attributed to the action of metabolites [e.g., hydrogen sulfide (H_2_S), short-chain fatty acids (SCFAs), trimethylamine, phospholipids], and biofilm (Zhou et al., 2024). In the context of increased concentrations of H_2_S, it was assumed that this may enhance nutrient intake as, on the one hand, H_2_S increases N-methyl-D-aspartic acid (NMDA) receptor activity (Zhou et al., 2024) and, on the other, activation of NMDA receptors elicits feeding in satiated rats, NMDA receptor antagonists block the eating elicited by NMDA, and, more importantly, NMDA blockade suppresses natural feeding and can reduce body weight (Stanley et al., 2011). SCFAc (including acetate, propionate, and butyrate) are microbial metabolites for gut-brain axis signaling, thereby also influencing food intake (O'Riordan et al., 2022). The significance of SCFAs is less conclusive for *Desulfovibrio* because, Hong et al. showed that DIO mice have indeed reduced fecal levels of SCFAs, but also that SCFAs correlate positively with *Desulfovibrio* (Hong et al., 2021), which seems somewhat contradictory since food intake is negatively influenced by SCFAs (Masse and Lu, 2023). However, we must concede that our findings do not provide any evidence for either mechanism as we did not determine fecal concentrations of H_2_S or SCFAs due to insufficient sample quantities and because measurement technology is lacking.

### Roles of desulvibrio for glucose regulation

Another important issue to address is whether a reduced abundance of *Desulfovibrio* should be associated with an improvement in glucose utilization. In 2013, a shotgun metagenome analysis showed changes in the composition and function of the gut microbiota in humans with normal, impaired, and diabetic glucose control (Karlsson et al., 2013). Such a link was confirmed in 2020 by metagenomic analysis, as prediabetes and T2DM were characterized according to changes in the gut microbiota and its functional genes, as well as by a decrease in the abundance of butyrate-producing bacteria (Wu et al., 2020). In this context, it seems particularly interesting that the proliferation of *Desulfovibrio* was strongly associated with a disturbance in glucose metabolism (Zheng et al., 2022; Kunasegaran et al., 2021). Moreover, mice treated with liraglutide, a GLP-1 receptor agonist approved for the treatment of T2DM, have been shown to induce recruitment of *Desulfovibrio* (Liu et al., 2020), thereby supporting the potential involvement of *Desulfovibrio* in glucose homeostasis through the production of H_2_S. Furthermore, treatment of DIO mice with cinnamon and grape pomace not only improved their obesity and glucose homeostasis but also altered the gastrointestinal microbiota (including reducing the abundance of *Desulfovibrio*), leading the authors to conclude that the improved metabolic situation correlates, at least in part, with the changes in the microbiota (Van Hul et al., 2018). As it was shown that H_2_S production by *Desulfovibrio* contributes to reducing butyrate concentration (Clark et al., 2021), butyrate activates peroxisome proliferator-activated receptor gamma (PPARγ) (Portincasa et al., 2024), and PPARγ activation increases insulin sensitivity (Soccio et al., 2014), we thus investigated whether the glucose homeostasis of the animals is also improved as a consequence of the altered microbiota as Desulfovibrio was increased by HFD feeding but also decreased in the mice that received fecal samples from TEL-pretreated donors. However, neither baseline glucose concentration nor insulin sensitivity was improved in the BL/6>f^TEL^ compared to the BL/6>f^VEH^ mice, which does not support a microbiota-dependent mechanism underlying the improvement in glucose homeostasis. In contrast, upon chronic oral TEL treatment, we have indeed seen improved glucose homeostasis and a reduction in insulin levels in TEL-treated rats with an altered microbiota in past studies in rats (Beckmann et al., 2021). However, in the cited study, as in the other reports using typical antidiabetic drugs such as metformin or liraglutide (Liu et al., 2020; Fernandez-Quintela et al., 2022), the question of the chicken or the egg remains unanswered. These studies indeed demonstrated an association between changes in the microbiota and improved glucose homeostasis, but not whether the improved glucose utilization is really causally linked with changes in the microbiota. We could not find any evidence for this causal link in the present study, which contradicts findings showing that FMT from lean healthy donors improves the insulin sensitivity of subjects with metabolic syndrome that were assumed to be driven by the recipient baseline fecal microbiota signature (Vrieze et al., 2012; Kootte et al., 2017). We did not analyze the microbiota of the donor mice in the present study; however, we still assume that it differed from healthy chow-fed controls, as we recently demonstrated, at least in rats, that network plots and random forest classifier differentiated between the taxonomic classification of chow-fed controls and HFE-fed/TEL-treated animals, although body weight and glucose homeostasis were similar in the two groups (Beckmann et al., 2021). Thus, the contradictory results indicated above seem plausible to us, even if the donor mice we used appeared to be of normal weight and obviously healthy (Huber et al., 2021). In addition, the fact that we conducted our study in non-ABx mice may have contributed to the fact that a reduced abundance of *Desulvibrio* may not have revealed this functional consequence of improved glucose homeostasis. Nonetheless, we do not doubt that TEL beneficially influences glucose homeostasis (Yang et al., 2013), but we are now more convinced that this is a coincidence and less a cause-and-effect principle. We are certain that TEL ameliorates glucose utilization, which has been shown several times in animal and human studies, especially since we have also carried out some work to elucidate the underlying mechanism. However, in addition to the widely accepted PPARγ-dependent mechanisms (Kintscher et al., 2008), we have also found evidence that insulin sensitivity is improved after ARB treatment independently of PPARγ regulation (Muller-Fielitz et al., 2012) and may be related more to the anti-inflammatory properties of these antagonists (Cole et al., 2010), normalized reactivity of the stress-axis (Miesel et al., 2012; Muller et al., 2007; Muller-Fielitz and Raasch, 2013; Raasch et al., 2006), or at least partly be attributed to a brain-related mechanism (Winkler et al., 2016). All these effects required chronic therapy using effective TEL doses.

In summary, we were able to show in the present study that FMT with stool samples from TEL-treated donor mice alters gastrointestinal microbiota with mainly a reduced abundance of *Desulfovibrio*, which was associated with a discrete weight loss but not with any improvement in glucose homeostasis. Whether this discrete weight loss, which is based on microbiome changes, effectively contributes to the therapeutic weight loss under TEL treatment remains an open question, but this does not seem quite as important to us, since the aim of the study was to determine whether the microbiome is altered by TEL and not what therapeutic effect a microbiome transfer has. A follow-up study in TEL-pretreated mice could, for example, investigate the significance of SCFA. Such a study would provide mechanistic evidence that TEL influences food intake and show how the microbiota is involved.

## Data Availability

Sequencing data were submitted to the European Nucleotide Archive (ENA) and can be accessed under accession number PRJEB75223.
