# Supplementary material for: Increase in body weight is lowered when mice received fecal microbiota transfer from donor mice treated with the AT1 receptor antagonist telmisartan
Source: Front Pharmacol. 2024 Nov 18;15:1453989. doi: 10.3389/fphar.2024.1453989 (PMC11608989; doi:10.3389/fphar.2024.1453989)
Supplement: Supplementary file 1 [file DataSheet1.docx]

Supplementary material

Increase in bodyweight is lowered when mice received fecal microbiota transfer from donor mice treated with the AT_1_ receptor antagonist telmisartan

Marco L. Freschi^1^, Axel Künstner^2^, Gianna Huber^1,3,4^ Ines Stölting^1^, Hauke Busch^2^, Misa Hirose^5^, Walter Raasch^1,3,4^

^1^Institute of Experimental and Clinical Pharmacology and Toxicology, University of Lübeck, Germany

^2^Medical Systems Biology Group, Institute of Experimental Dermatology, University of Lübeck, Germany

^3^DZHK (German Centre for Cardiovascular Research), Partner Site Hamburg/Kiel/Lübeck, Lübeck, Germany

^4^CBBM (Center of Brain, Behavior and Metabolism), Germany

^5^Institute of Experimental Dermatology, University of Lübeck, Germany

Additional discussion on the question of whether residual telmisartan contributed to the effects gained through stool transplantation

Telmisartan (TEL) concentrations were not measured in the stool samples used for the fecal microbiota transfer (FMT) in this study; however, we are nevertheless convinced that direct TEL effects after reabsorption did not contribute to the weight-regulatory effects, as the stool samples were highly diluted for preparation for gavage. This assumption is based on the following considerations and calculations. The obese donor animals treated with TEL had an average body weight of approximately 30 g and were treated with a dose of 8 mg/kg^bw^ TEL daily (1). Assuming that TEL was excreted at steady state equilibrium, it can be assumed that the amount of TEL excreted is equal to the amount of drug ingested per day. A HFD-fed mouse excretes approximately 2.5 g of feces per day (2). Based on these assumptions, the TEL concentration in the stool samples is approximately 0.1 mg/g^feces^. During preparation (see methods section), a dilution by a factor of 100 was carried out and 150 µl of the suspension was then administered 3 times per week. With an average body weight of the test animals subjected to FMT of approx. 40 g (1), this resulted in a daily TEL dose of approx. 0.0016 mg/kg^bw^. Despite all the necessary simplifications in this calculation, it is quite clear that the potentially administered amount of TEL is approx. 5,000 times smaller than that used in previous studies.

Fig. S1: Study protocol: Seven weeks after initiating a high-fat diet (HFD), C57BL/6N mice received fecal microbiota for 8 weeks from donor mice by oral gavage, continuing HFD feeding. Stool samples came from mice that were treated with TEL (8mg/kg/d by gavage, 12 weeks). Controls received feces samples from vehicle/HFD-treated obese mice (BL/6>f^VEH^). The body weight was regularly monitored by weighing the animals. The mice were subjected to laboratory-established functional tests during the intervention, namely, indirect calorimetry at d70-d77 using the Phenomaster System (TSE, Germany), body composition measurement by using the Minispec BCA analyzer (LF-110, Bruker) (at d92), and an insulin tolerance test (ITT) at d84. Stool samples were collected at three time points for microbiota analysis: 0, 49, and 101 days. Microbiota of the stool samples from these acceptor mice was analyzed by 16S rRNA gene amplicon sequencing.

Fig. S2: Quotient between daily energy consumption and daily energy intake in BL/6>f^VEH^ or BL/6>f^TEL^ mice; means±SD, n=4-6. The statistical comparison between the two groups was determined using Wilcoxon analysis.

Fig. S3: Phylum abundance at d0 and d49. At time point d0, the mice were still receiving chow diet, while d49 represents the time point immediately before FMT, when the two groups of mice had been fed HDF for 7 weeks but had not yet received microbiome transfer.

Fig. S4: Family abundance at d0 and d49. At time point d0, the mice were still receiving chow diet, while d49 represents the time point immediately before FMT, when the two groups of mice had been fed HFD for 7 weeks but had not yet received microbiome transfer.

Fig. S5: Genus abundance at d0 and d49. At time point d0, the mice were still receiving chow diet, while d49 represents the time point immediately before FMT, when the two groups of mice had been fed HDF for 7 weeks but had not yet received microbiome transfer.

Fig S6: Correlation analyses according to Pearson between final body weight and different phyla. The gray and red lines represent the best-fit lines of the correlation analyses of the BL/6>f^VEH^ or BL/6>f^TEL^ mice. Pearson R and P values are presented in Fig. 8.

Fig S7: Correlation analyses according to Pearson between final body weight and different families. The gray and red lines represent the best-fit lines of the correlation analyses of the BL/6>f^VEH^ or BL/6>f^TEL^ mice. Pearson R and P values are presented in Fig. 8.

Fig S8: Correlation analyses according to Pearson between final body weight and different genera. The gray and red lines represent the best-fit lines of the correlation analyses of the BL/6>f^VEH^ or BL/6>f^TEL^ mice. Pearson R and P values are presented in Fig. 8.

Tab S1: Comparison between the abundances of certain genera before and after 49 days of HFD feeding of the mice

| genus | d0 | d49 | P value | fold change |
| --- | --- | --- | --- | --- |
| *Anaerotignum* | 1.7 ± 1.1 | 176.6± 82.5 | 0.00016 | +106 |
| *Desulfovibrionia_uncl* | 9.5 ± 5.0 | 924.3 ±471.7 | 0.00016 | +97 |
| *Faecalibaculum* | 8.7 ± 8.8 | 839.6 ±584.1 | 0.00016 | +97 |
| *Acetatifactor* | 5.1 ± 4.4 | 451.83±227.23 | 0.00016 | +89 |
| *Kineothrix* | 20.5 ± 19.5 | 1584.8 ± 998.5 | 0.00016 | +77 |
| *Acutalibacteraceae_uncl* | 24.8 ± 27.9 | 1758.7 ± 1740.0 | 0.00016 | +71 |
| *Bacteroidales* | 107.4 ± 35.1 | 4003.1 ± 2544.9 | 0.00016 | +37 |
| *Lawsonibacter* | 55.4 ± 37.9 | 1710.1± 428.3 | 0.00016 | +31 |
| *Parabacteroides* | 3.3 ± 2.1 | 57.50 ± 38.9 | 0.00016 | +18 |
| *Ruminococcaceae_uncl* | 58.0 ± 28.9 | 941.7 ±369.9 | 0.00016 | +16 |
| *Ligilactobacillus* | 197.2 ± 73.8 | 927.2 ± 321.3 | 0.00016 | +5 |
| *Desulfovibrio* | 112.5 ± 42.4 | 404.3 ± 283.1 | 0.0064 | +4 |
| *Paramuribaculum* | 562.8 ±189.2 | 53.2 ± 43.8 3 | 0.00016 | -11 |
| *Duncaniella* | 1280.1 ± 319.8 | 60.2 ± 40.4 | 0.00016 | -21 |
| *Turicimonas* | 681.7 ± 231.8 | 6.0 ±5.8 | 0.00016 | -114 |
| *UBA7050* | 121.6 ± 151.1 | 0.1 ±0.0 | 0.00016 | -1216 |

Reference List

1. Huber G, Ogrodnik M, Wenzel J, Stölting I, Huber L, Will O, et al. Telmisartan prevents high-fat diet-induced neurovascular impairments and reduces anxiety-like behavior. Journal of cerebral blood flow and metabolism : official journal of the International Society of Cerebral Blood Flow and Metabolism. 2021;41(9):2356-69.

2. Kraus D, Yang Q, Kahn BB. Lipid Extraction from Mouse Feces. Bio-protocol. 2015;5(1).
